# From biofilms to fibrosis: recent advances in research on the pathological microenvironment linked to ureteral stents and their surface functionalization

**DOI:** 10.3389/fbioe.2026.1925047

**Published:** 2026-09-28

**Authors:** Yiru Wang, Qing Yang, Yizhe Lv, Xiao Li, Yaoqin Xue

**Affiliations:** 1 College of Medical Imaging, Shanxi Medical University, Taiyuan, China; 2 Department of Interventional Therapy, Shanxi Province Cancer Hospital/ Shanxi Hospital Affiliated to Cancer Hospital, Chinese Academy of Medical Sciences/Cancer Hospital Affiliated to Shanxi Medical University, Taiyuan, China; 3 School of Public Health, Shanxi Medical University, Taiyuan, China; 4 Third Clinical College, Shanxi University of Chinese Medicine, Jinzhong, China; 5 School of Basic Medical Sciences, Shanxi Medical University, Taiyuan, China

**Keywords:** biofilm, clinical translation, encrustation, fibrosis, functional modification, pathological microenvironment, ureteral stent

## Abstract

In the realm of urology, ureteral stents serve as common implantable devices. However, their prolonged use is often associated with complications, including biofilm infections, encrustation, chronic inflammation, and ureteral fibrosis, which significantly affect patients’ quality of life and reduce clinical efficacy. Growing evidence has shown that the development and progression of these problems are not isolated events but the consequence of an imbalance of the pathological stent microenvironment under the influence of multiple factors. Therefore, precise regulation of the stent microenvironment has attracted much attention in the design of next-generation ureteral stents. In this study, we provide a detailed investigation into the mechanisms that lead to the creation of a pathological state after ureteral stents are installed, with particular emphasis on the interactions and regulatory mechanisms associated with stent-related problems. Recent strategies, for functional surface modification, including anti-encrustation, antibacterial, anti-inflammatory, and antifibrotic methods are reviewed, and the mechanisms of action as well as research progress of various novel coatings and functional materials are discussed. Meanwhile, based on the available clinical research information, we objectively analyze the barriers in the translation of functionalized ureteral stents.

## 1 Introduction

Ureteral stents are prevalent and essential medical devices in urology, and they efficiently alleviate obstruction and are indispensable in critical clinical situations, including the management of urinary stones and the alleviation of upper urinary tract obstruction (Matsuura et al., 2019; Ordonez et al., 2019; Sali and Joshi, 2020). The prolonged placement of ureteral stents, acting as an external foreign body, unavoidably alters the normal ureteral environment, leading to many associated problems. Research indicates that as many as 80% of patients encounter problems of differing severity with stent insertion, predominantly comprising bladder irritation, hematuria, stent encrustation or stone formation, and urinary tract infections. Lower urinary tract symptoms are the most widespread, affecting between 32% and 80% of individuals (Geavlete et al., 2021; Ooi et al., 2026). These issues not only diminish patients’ quality of life but may also advance to severe adverse events, such as irreversible renal impairment and urinary sepsis, thereby jeopardizing patients’ lives (Harper et al., 2022; Wang et al., 2024) (Figure 1). From a pathophysiological point of view, ureteral stent placement can initiate a series of intricate cascading reactions, resulting in the progressive development of a pathological microenvironment surrounding the stent, characterized by a conditioning film, biofilm, crystal deposits, inflammatory mediators, and aberrant mechanical stimuli. In this context, numerous problems interrelate and intensify each other (Table 1). Consequently, precise control of the diseased microenvironment is an important strategy for preventing and managing problems at their source. It can also improve the performance of ureteral stents.

**FIGURE 1:**
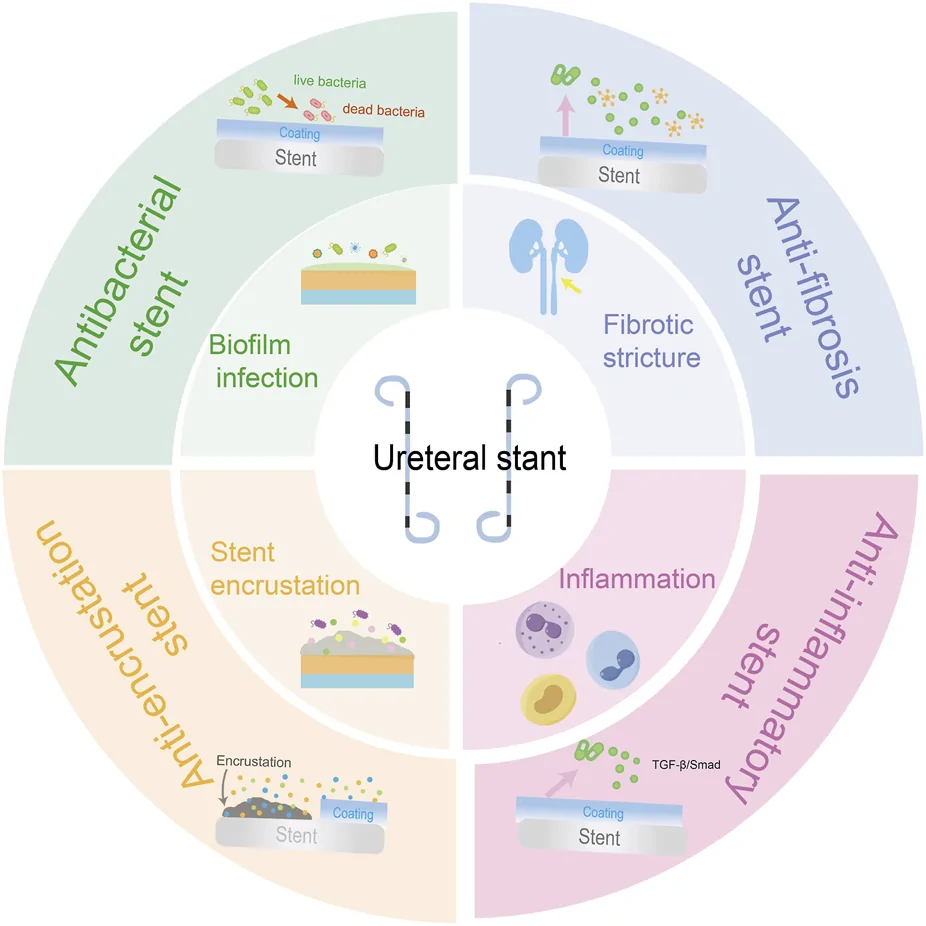
Ureteral stent-related complications and corresponding functionalization strategies.

**TABLE 1:** Major complications associated with ureteral stents and their underlying mechanisms.

Stent-associated complications	Main pathological mechanisms	Key biological processes and signaling pathways	Clinical manifestations and impacts	Potential functional modification strategies
Biofilm formation and persistent infection	The stent surface serves as a platform for bacterial adhesion and colonization, promoting the formation of extracellular polymeric substances (EPS) and mature biofilms	Initial bacterial adhesion, quorum sensing, EPS secretion, and antimicrobial tolerance; regulation of bacterial adhesion-related proteins and biofilm-associated genes	Urinary tract infection, bacteriuria, failure of antibiotic therapy, and increased risk of stent-associated infections	Antimicrobial coatings, antimicrobial peptides, metal ion-based coatings, antibiofilm surface modifications, and controlled antimicrobial release systems
Encrustation and crystal deposition	Mineral ions in urine undergo nucleation, deposition, and crystal growth on the stent surface	Protein adsorption, bacteria-induced urinary pH alteration, calcium oxalate and calcium phosphate deposition, and mineral crystal formation	Stent obstruction, difficult stent removal, ureteral blockage, and potential renal function impairment	Hydrophilic coatings, anti-adhesive materials, zwitterionic polymers, and strategies to reduce protein/crystal adhesion
Inflammatory response	The stent acts as a foreign body that induces local immune responses and creates a pro-inflammatory microenvironment	Toll-like receptor (TLR) activation, NF-κB/MAPK signaling pathways, inflammatory cytokine release (e.g., IL-6 and TNF-α), and oxidative stress (ROS generation)	Lower urinary tract symptoms, pain, hematuria, and chronic inflammatory tissue injury	Anti-inflammatory drug-loaded coatings, immunomodulatory materials, and ROS-responsive smart coatings
Fibrotic remodeling and ureteral stricture	Persistent inflammation and mechanical irritation induce fibroblast activation and extracellular matrix deposition	TGF-β/Smad signaling pathway activation, fibroblast activation, collagen deposition, and epithelial–mesenchymal transition (EMT)	Ureteral wall thickening, luminal narrowing, urinary obstruction, and long-term impairment of urinary drainage	Antifibrotic drug delivery systems, TGF-β pathway regulation, and tissue-regenerative biomaterials
Mechanical irritation and stent-related symptoms	Long-term contact between the stent and urinary tract mucosa causes mechanical friction and local irritation	Urothelial injury, neural stimulation, and local inflammatory mediator release	Urinary frequency, urgency, pain, hematuria, and reduced quality of life	Flexible material design, low-friction surfaces, and smart material-based mechanical optimization

Presently, conventional polymer stents, such as silicone and polyurethane stents, are widely used in clinical practice; however, their surfaces are prone to biofilm formation and mineral salt deposition, which can easily lead to infection and stent encrustation (Mathias and Wiseman, 2022; Guo and Yuan, 2023). Despite the advantages of metal stents, including great durability, their installation in practice is quite challenging, and long-term retention poses hazards of stent fracture and tumor tissue regeneration (Lugmayr and Pauer, 1996; Chen Y. et al., 2025). Recent innovative designs, such as biodegradable and magnetic stents, have attracted considerable attention for their potential to solve clinical management problems. They may reduce complications during stent removal and reduce accidental long-term retention. However, these technologies are still at the stages of basic research and preliminary clinical investigation (Damodaran et al., 2023; Li J et al., 2023; Khan et al., 2025). Despite advancements in materials science and manufacturing technology that have facilitated the ongoing optimization of ureteral stents regarding material selection and structural design, the currently available clinical stents predominantly provide passive support and drainage, thereby hindering the active regulation of the stent microenvironment and the prevention of complications at their origin (Lee et al., 2021).

Prior reviews have provided an overview of advances in the research of ureteral stent materials, surface modifications, and antimicrobial strategies. However, although these studies have focused primarily on enhancing the material properties and functional performance, there is a notable lack of comprehensive discussion about the intricate biological phenomena triggered by the introduction of ureteral stents and their contribution to the initiation and development of complications. Nevertheless, the pathological microenvironment of ureteral stents is crucial to decipher the long-term effectiveness of stents and guide the development of next-generation devices. The purpose of this review is to describe the pathological mechanisms underlying ureteral stents and their potential roles in the formation of major complications, viewed from the perspective of modulating the pathological microenvironment. This review also covers recent advances in the functionalization of ureteral stent surfaces, such as antistone, antimicrobial, anti-inflammatory, and smart-responsive technologies, focusing on their mechanisms of action, functional benefits, and potential clinical applications. The purpose of this review is to put forward a research framework that integrates the pathological mechanisms related to ureteral stents and the methods of materials engineering to provide a theoretical basis and technical guidance for the design, development, and potential clinical application of novel ureteral stents with active regulatory functions.

## 2 Literature search strategy

A literature search was conducted to identify recent advances in the pathological microenvironment associated with ureteral stents and surface functionalization strategies. Electronic databases, including PubMed and Web of Science, were searched for relevant studies published between 2021 and 2026. The search strategy incorporated terms related to ureteral stents (e.g., “ureteral stent” and “ureteric stent”), stent-associated complications (e.g., “biofilm,” “encrustation,” “inflammation,” and “fibrosis”), and surface functionalization approaches (e.g., “surface functionalization,” “coating,” “antibacterial,” “anti-inflammatory,” and “smart-responsive materials”). Reference lists of relevant articles were also manually screened to identify additional studies.

Inclusion criteria: We included studies investigating pathological mechanisms associated with ureteral stents (e.g., biofilm formation, bacterial colonization, calcification, inflammatory responses, fibrosis, and biomaterial interactions) and ureteral stent materials, biodegradable stents, surface modifications, antimicrobial coatings, anti-inflammatory improvements, drug delivery systems, and smart-responsive materials. Original studies, clinical trials, experimental research, and relevant review articles published in the English language from March 2021 to March 2026 were considered. We excluded irrelevant papers on ureteral stents, those exclusively focusing on surgical techniques without discussing stent-related mechanisms, those with low relevance to the topic of this review, conference abstracts, editorials, letters to the editor, and duplicate publications. A schematic overview of the literature search process was provided (Figure 2).

**FIGURE 2:**
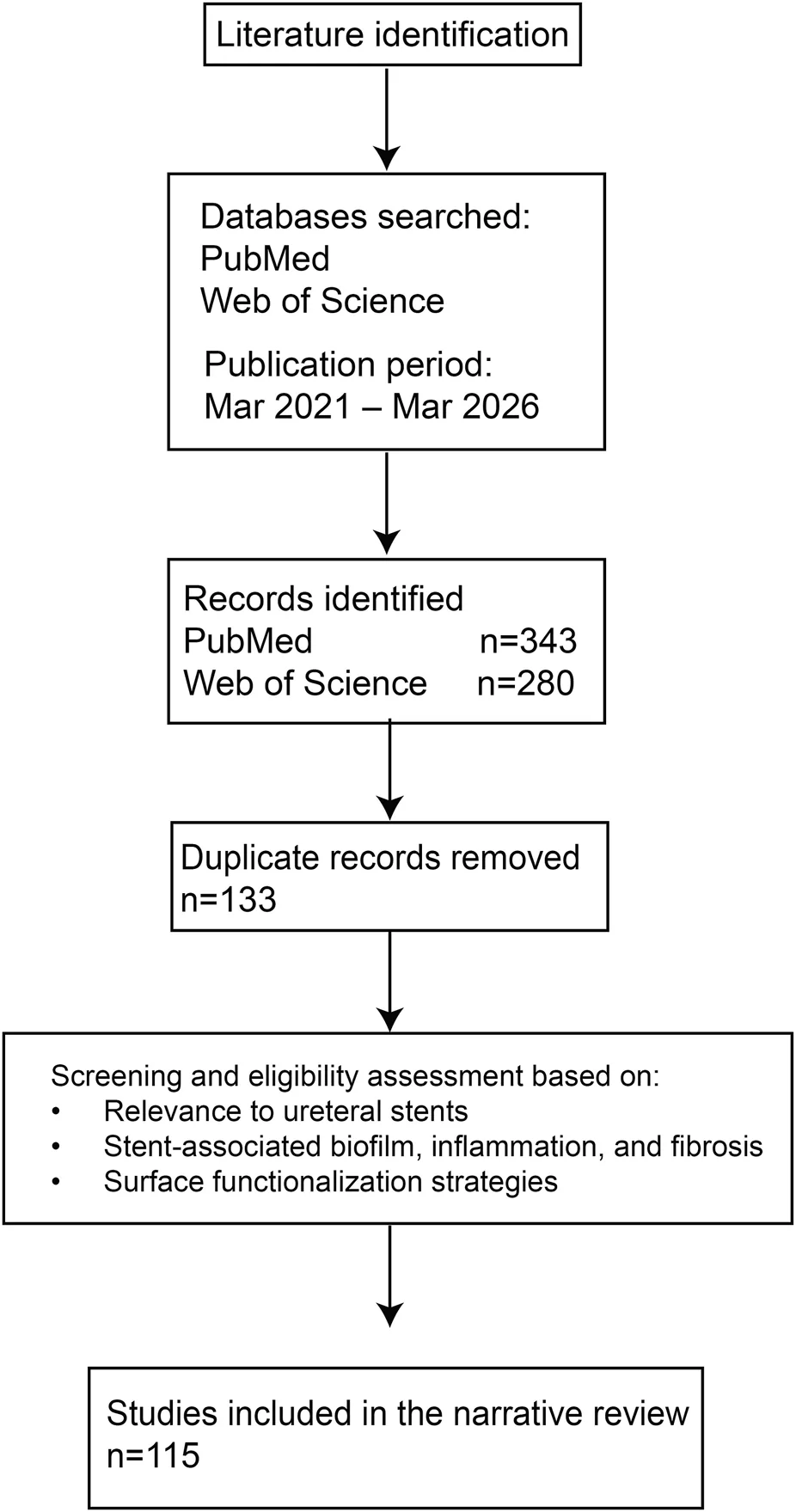
Flow diagram of literature identification, screening, and the inclusion process.

## 3 Pathological microenvironment associated with ureteral stents: mechanisms underlying major complications

### 3.1 Conditioning film formation and microbial colonization

The introduction of ureteral stents into the urinary tract leads to the rapid adsorption of macromolecules, including Tamm–Horsfall protein (THP), polysaccharides, and lipids, onto the stent surface by physicochemical forces such as hydrophobic interactions, electrostatic interactions, and van der Waals forces. This results in the formation of a dense layer of film named the “conditioning film” (Scotland et al., 2020; Bhagwat et al., 2021a). The formation of the conditioning film markedly changes the original surface properties of the material and provides adhesion sites and a nutrient-rich environment for ensuing bacterial attachment and biofilm growth (Bhagwat et al., 2021b).

### 3.2 Biofilm formation and persistent infection

A biofilm is a structured microbial community that forms when microorganisms such as bacteria and fungi attach to the surface of stents and secrete extracellular polymeric substances (EPS). Its main functions are to provide the structure and to protect the microbial community from outside interference. Biofilms are important in the initiation and progression of ureteral stent-related issues (Scalia and Najmi, 2025).

#### 3.2.1 Process of biofilm formation

After the conditioning film forms, bacteria can achieve reversible adhesion by van der Waals forces, hydrophobic contacts, and additional processes (Senevirathne et al., 2022). Bacteria utilize features such as flagella or fimbriae to increase the binding strength by forming specific bonds with ligands in the conditioning film (Wang Z et al., 2026). This leads to irreversible attachment. For instance, *Escherichia coli* uses the FimH adhesin on its fimbriae to selectively recognize and bind to mannose residues on THP (Dahlberg et al., 2022; Guedes et al., 2026). Wall shear stress (WSS) is also another important factor in this process (Amado et al., 2023). Environments with low WSS facilitate initial bacterial adherence, while those with high WSS can remove bacteria that have not yet formed strong, irreversible adhesion (Simões et al., 2022; Hubert et al., 2024). After irreversible adhesion occurs, a certain WSS may enhance adhesion strength by triggering conformational alterations in bacterial adhesion proteins, and this process is referred to as the “catch bond” mechanism (Thomas et al., 2022).

Bacteria that have irreversibly attached multiply by binary fission, microcolony formation, and large amounts of EPS secretion. EPS consist of polysaccharides, proteins, lipids, and other constituents (Lu et al., 2024) and are essential for preserving the integrity of biofilms. The 3D network structure of EPS is a good barrier to prevent the penetration of harmful chemicals such as antimicrobial agents and toxins, and it can also provide mechanical support to the bacteria contained therein. In addition, EPS help with the water balance of cells and the regulation of osmotic pressure, therefore protecting the structural integrity and physiological homeostasis of the biofilm (Chai et al., 2024; Wang Y et al., 2026). At this stage, bacteria can control the regulation of genes related to collective actions through quorum sensing (QS), which not only regulates the synthesis and secretion of EPS but also participates in the regulation of bacterial metabolism and phenotypic transformation (Chang et al., 2022).

Then, large amounts of the EPS matrix are excreted and interspersed among the bacteria, which act as water channels for nutrition transfer and metabolic waste elimination (Afrasiabi and Partoazar, 2024). At the same time, bacteria experience metabolic differentiation, culminating in the formation of a highly organized and functionally differentiated three-dimensional spatial structure (Barajas-García et al., 2023; Pan et al., 2023). In the end, cells are actively shed from established biofilms, return to a motile state, and disperse into new environments. This process is governed by signals including quorum sensing, oxidative stress, and nutrient depletion and can be enhanced by the production of EPS-degrading enzymes that release free-swimming bacteria (Wen et al., 2022; Kauser et al., 2023). These bacteria may be new sources of infection, leading to the formation of new biofilms in other areas of the stent or entry into urine, causing problems such as urinary tract infections (Ma et al., 2023) (Figure 3A).

**FIGURE 3:**
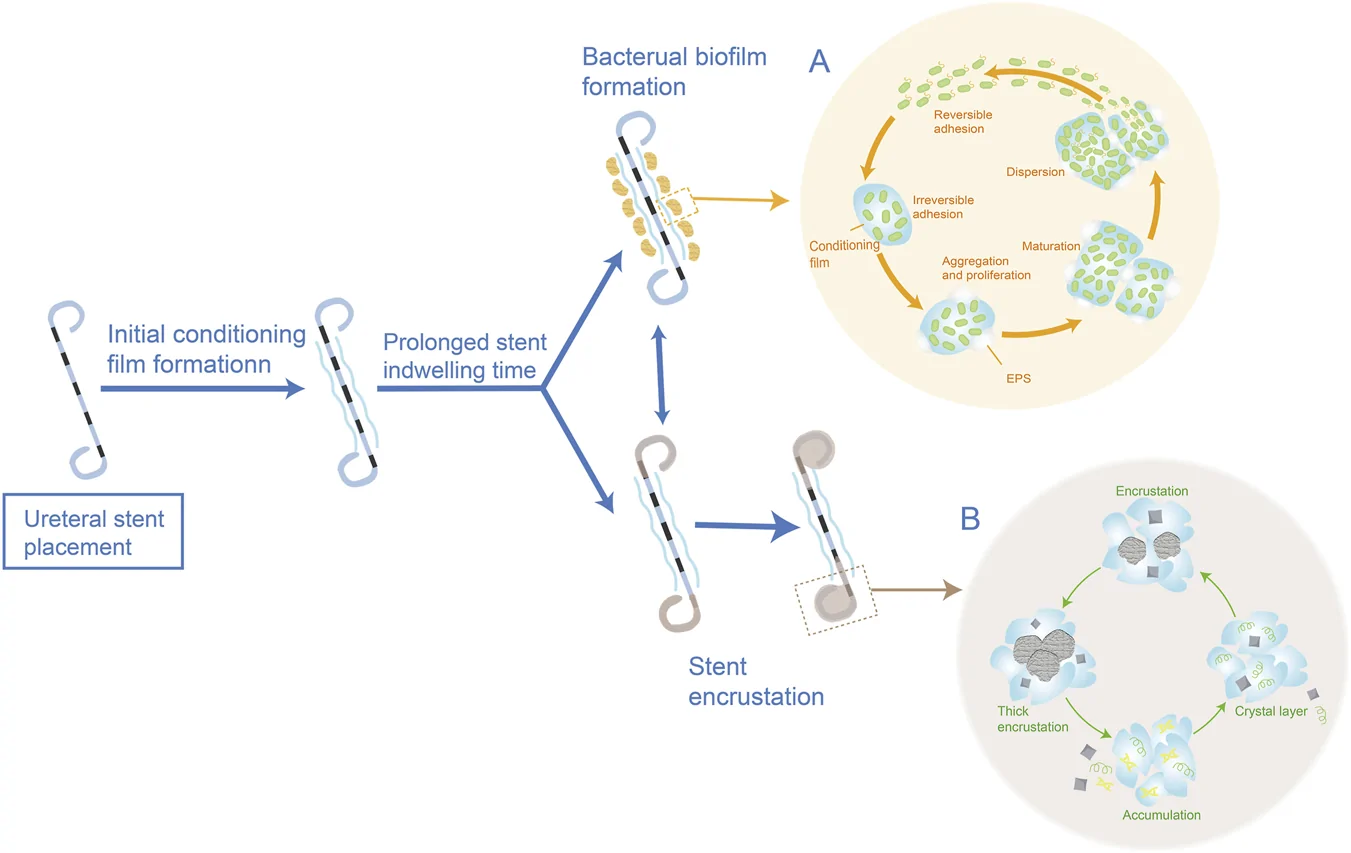
Biofilm **(A)** and encrustation formation **(B)** after ureteral stent implantation.

#### 3.2.2 Mechanisms of persistent infection

Microbial attachment and colonization can occur soon after the insertion of a ureteral stent, and the biofilm that develops on the surface of the stent is thought to be a major factor in the persistence of infection and increased risk of stent-related complications (Buhmann et al., 2018; Cornette et al., 2024). Research indicates that the bacterial colonization rate after ureteral stent insertion varies between 42% and 90% (Abu et al., 2022). About 54% of colonized cases advance to clinically identifiable urinary tract infections, and there exists a substantial positive connection between the stent period and infection risk (Ortolini et al., 2025). *Escherichia coli*, Enterobacteriaceae, *Pseudomonas aeruginosa*, and other Gram-negative bacteria are the principal pathogenic agents (Li Y et al., 2023; Mousavi et al., 2024). The antibiotic resistance of biofilm-associated bacteria can exceed that of free-floating bacteria by over 1,000 times (Deng et al., 2025).

Multidrug resistance of infections is not only a consequence of any single factor but also a result of the totality of the effects of numerous pathogenic factors. First, the EPS matrix and the dense structure of the biofilm create a diffusion limitation effect, which limits the diffusion of antibiotics into deeper locations, leading to a drastic reduction in the local effective drug concentration. For example, the negatively charged EPS matrix can entrap positively charged aminoglycoside drugs (Coppens et al., 2023; Tian et al., 2023). Second, persister cells located in the deeper layers of the biofilm exist in a state of low metabolic dormancy owing to a lack of oxygen and nutrients. This renders them impervious to drugs intended for actively proliferating bacteria, thereby obstructing the attainment of optimal sterilization (Hindieh et al., 2025). Furthermore, acidic metabolic products generated by bacterial metabolism, including hydrolases, can deactivate specific antibiotics. For instance, β-lactamases can inactivate β-lactam antibiotics (Dashtbani-Roozbehani and Brown, 2021; Lu et al., 2023). Bacteria in biofilms can greatly upregulate the expression of genes related to the multidrug efflux pump to reduce the intracellular concentration of antibiotics through active efflux mechanisms below the inhibitory concentration. In addition, the biofilm environment can induce stress responses in bacteria that can result in a transitory drug-tolerant condition, therefore promoting the overall survival capacity of the bacterial population. These multilayer mechanisms act in concert to enable biofilm-encapsulated bacteria to survive conventional antibiotic treatment, leading to recurrent stent-related infections and posing a major challenge to clinical antimicrobial therapy (Yang et al., 2023).

### 3.3 Stent encrustation and crystal deposition

Stent encrustation is a process whereby ions in urine, such as Ca^2+^, C_2_O_4_^2−^, and PO_4_^3−^, undergo heterogeneous nucleation, crystal growth, and aggregation on the surface of the stent, resulting in crystalline deposits, which leads to stent blockage and impaired renal function. In addition, build-up on the stent surface could make it difficult to remove the stent. Studies have shown a strong positive relationship between the retention period of stent installation and the incidence of stent encrustation. The incidence is around 9.0%–9.2% after 6 weeks of placement, increases to 48%–57% between 6 and 12 weeks, and may reach as high as 76%–77% after more than 12 weeks (Maxim et al., 2025).

The conditioning film poststent implantation supplies a binding interface and key sites for crystal nucleation. When the concentrations of urinary ions—such as Ca^2+^, C_2_O_4_^2−^, and PO_4_^3−^—exceed the solubility limit and the solution becomes supersaturated, these ions initially arrange themselves in an ordered manner on the active sites of the conditioning film to form critical crystal nuclei. As urine continues to flow, crystals continue adsorbing more ions, which grow larger and form a crust (Hayashi et al., 2024) (Figure 3B). The biofilm creates an optimal environment for bacterial adhesion and proliferation. Urease, produced by urease-producing bacteria such as *Proteus mirabilis*, degrades urea, resulting in substantial ammonia production and a significant increase in local urine pH to alkaline levels. This substantially enhances the nucleation of magnesium ammonium phosphate and calcium phosphate crystals, resulting in the development of a characteristic infectious crust (De Grazia et al., 2020). In the biofilm, the EPS matrix acts as an organic template for heterogeneous nucleation of crystals, resulting in a synergistic amplification effect with the urease-induced increase in pH. In addition, an unusual WSS distribution is an important mechanical aspect that leads to encrustation. Research has shown that more than 60% of the lateral orifices in the stent are located in low WSS locations, which is the region more likely to retain, aggregate, and stably deposit crystal particles (Mosayyebi et al., 2022).

### 3.4 Stent-related inflammatory response and ureteral fibrotic stricture

After a stent—an external foreign body—is inserted into the body, the host’s immune system consistently reacts abnormally. As the inflammatory response progressed from the early acute phase to the chronic phase, luminal stricture and ureteral fibrosis eventually resulted (Vogt and Chokri, 2020). This process can cause structural damage to the ureteral wall and progressive narrowing of the ureteral lumen, eventually leading to recurrent urinary tract obstruction, worsening hydronephrosis, and even irreversible renal dysfunction. It also causes physical discomfort in patients, such as pain in the lower back and abdomen and bladder irritation. The effect of ureteral stents on fibrosis is complex and may depend on the type and degree of ureteral injury. Some recent studies suggest that ureteral stents protect against fibrosis in some injury models by promoting injury repair and decreasing fibrotic response (Gu et al., 2025).

#### 3.4.1 Initiation of inflammatory responses

The biofilm that develops following stent implantation is abundant in different chemokines and cytokines that can selectively attract inflammatory cells such as neutrophils and monocytes, starting the acute inflammatory phase (Barbeck and Jung, 2026). This stage, characterized by congestion and edema of the ureteral mucosa, rapid neutrophil infiltration, and a brief, massive release of inflammatory mediators, serves as the host’s primary defense against foreign body invasion. This defensive reaction is abnormally and persistently activated after long-term stent installations, which leads to chronic inflammation.

During the chronic inflammatory phase, fibrinogen and lipopolysaccharide (LPS) on the stent surface constantly activate the Toll-such as receptor 4/MyD88 pathway. This stimulates nuclear factor kappa B (NF-κB) signaling and promotes the cascade release of pro-inflammatory molecules such as TNF-α, interleukin-1 beta (IL-1β), and interleukin-6 (IL-6). Macrophage infiltration and altered M1/M2 polarization contribute to a persistent inflammatory state via the Janus kinase/signal transducer and activator of transcription pathway (Rahmati et al., 2020). In this phase, abnormal wall shear stress (WSS) induces Ca^2+^ influx through mechanosensitive channels such as Piezo1 and TRPV4, converting mechanical stimuli into inflammatory signals and promoting local inflammation (Swain and Liddle, 2021; Huang et al., 2023). Moreover, ischemia, biofilm toxin release, and mechanical friction and tension from the stent can all increase inflammation and tissue damage. The transforming growth factor beta-1 (TGF-β1)/Smad signaling axis is triggered when chronic inflammation worsens, causing a slow shift from inflammation to fibrosis.

#### 3.4.2 Ureteral fibrosis and luminal stenosis

The most important long-term adverse consequences after ureteral stent placement are ureteral fibrosis and luminal stricture. Chronic inflammation, mechanical irritation, ischemia–hypoxia, and multiple profibrotic pathways result in urothelial–mesenchymal transition (UMT), excessive extracellular matrix (ECM) deposition, and irreversible remodeling of the ureteral wall, resulting in luminal stricture or even occlusion (Reicherz et al., 2023).

Local TGF-β1 expression is markedly elevated because of chronic inflammation. Binding of TGF-β1 to its receptor induces the phosphorylation of Smad2/3. The phosphorylated Smad2/3 then forms a complex with Smad4 and translocates into the nucleus, directly triggering the transcription of extracellular matrix genes, including COL1A1, COL3A1, and fibronectin (Chen F et al., 2025). Conversely, non-Smad pathways, including mitogen-activated protein kinase (MAPK), p38, c-Jun N-terminal kinase (JNK), PI3K/AKT, and ROCK, collaboratively enhance fibroblast proliferation, migration, and anti-apoptotic mechanisms (Yang et al., 2022). In addition, prolonged compression by the scaffold leads to local tissue ischemia and hypoxia, as well as aberrant activation of the renal renin–angiotensin system (RAS) and mTOR pathway, which further aggravates fibroblast activation and ECM deposition. The persistent effects of the abovementioned inflammation and mechanical stress induce the transition of circulating fibroblasts to myofibroblasts. This results in a dysregulation of extracellular matrix remodeling with a build-up of excess collagen and a reduction in degradation. At the same time, some of the damaged urothelial cells may undergo UMT, increasing the availability of myofibroblasts and accelerating the process of fibrosis (Shi et al., 2023) (Figure 4).

**FIGURE 4:**
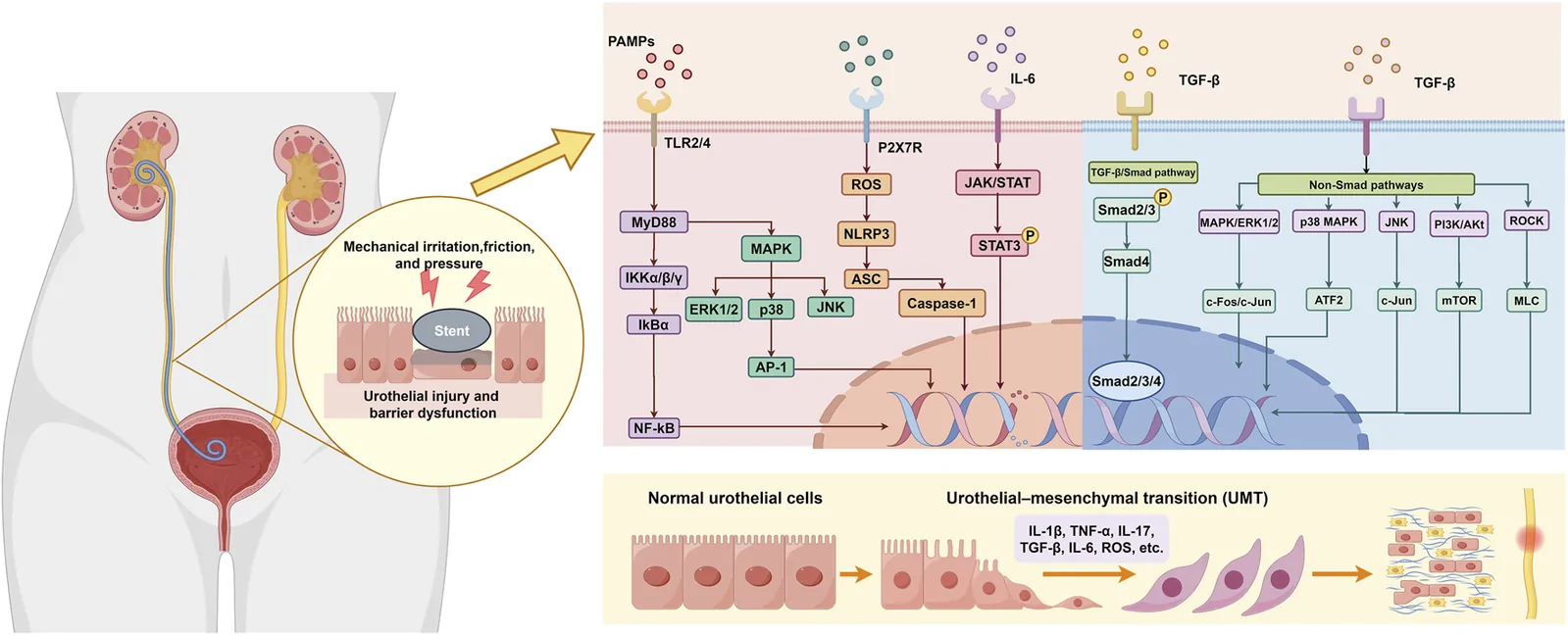
Mechanisms of inflammation and fibrosis associated with ureteral stents. This image was created by Figdraw (ID:TPPTI5a25f). Abbreviations: IL-6, interleukin-6; TGF-β, transforming growth factor beta; NF-κB, nuclear factor kappa B; MAPK, mitogen-activated protein kinase; ERK1/2, extracellular signal-regulated kinase 1/2; JNK, c-Jun N-terminal kinase; AP-1, activator protein-1; NLRP3, NOD-, LRR-, and pyrin domain-containing protein 3; JAK/STAT, Janus kinase/signal transducer and activator of transcription pathway; mTOR, mechanistic target of rapamycin.

## 4 Surface functionalization strategies for modulating the pathological microenvironment of ureteral stents

### 4.1 Anti-encrustation strategies

Conventional anti-encrustation surface modification techniques predominantly depend on crystallization inhibitors or drug-releasing coatings. But these methods are hindered by limitations such as fast depletion, electrolyte disruption, and the potential for long-term drug resistance. Conversely, the zwitterionic groups in zwitterionic materials interact with adjacent water molecules via ion–dipole interactions, creating a robust hydration layer on the scaffold surface that diminishes contact between bacteria, ions, and the scaffold, thus effectively preventing bacterial adhesion and biofilm formation on the scaffold surface (Qin et al., 2025).

Sulfobetaine methacrylate (SBMA) is a representative amphoteric functional monomer commonly utilized in the formulation of antifouling and highly biocompatible polymer coating systems (Zhu et al., 2021). Wu et al. (2026) employed the hydration effect of SBMA to develop a versatile zwitterionic polymer layer applied to the polyurethane stent (PU/ATS). This scaffold successfully prevented the joining of scaling mineral ions for as long as 90 days and markedly hindered the sticking of prevalent urinary tract bacteria, including *Escherichia coli*, *Staphylococcus aureus*, and *Proteus mirabilis*. Trimethylamine N-oxide (TMAO) represents a new class of zwitterionic materials. Their unique structure involves a positively charged nitrogen atom (N^+^) directly linked to a negatively charged oxygen atom (O^−^). This interaction with neighboring water molecules results in the formation of a stable hydration layer (Huang et al., 2021). Li et al. (2026) formulated a coating (PU/3-(trimethoxysilyl)propyl methacrylate (TMSPMA)/poly(trimethylamine N-oxide) (pTMAO), referred to as PTO) employing a superwetting-assisted interfacial polymerization (SAIP) approach in conjunction with dip-coating technology, utilizing poly(trimethylamine N-oxide) (pTMAO) and 3-(trimethoxysilyl)propyl methacrylate (TMSPMA) as precursors. Experimental results indicated that the PTO scaffold has a contact angle of 0°, demonstrating superhydrophilicity, and displayed considerable antibacterial adhesion effects against prevalent urinary tract infections, including *Escherichia coli* and *Proteus mirabilis*. Moreover, following the placement of the PTO scaffold, an *in vitro* model of dynamic urine lasting between 30 and 90 days, scale deposition within 90 days was markedly inferior to that of PU and PT scaffolds, indicating superior anti-encrustation efficacy.

Despite the impressive performance of amphoteric coatings in artificial and natural settings, their clinical use faces several challenges. These coatings mainly provide anti-adhesion properties and are ineffective in eliminating pre-existing biofilms or adherent bacteria. In patients with severe infections or those at high risk, a singular amphoteric approach may be inadequate to prevent the advancement of infectious encrustation (Roh et al., 2025) (Table 2).

**TABLE 2:** Comparison of functionalization strategies for ureteral stents.

Regulatory strategies	Primary targets	Representative materials/technologies	Mechanism of action	Highest level of validation achieved	Advantages	Limitations
Anti-encrustation strategies	Crystal nucleation and deposition processes	Heparin, PTO, TMAO, SBMA, and MPC	Inhibit protein adsorption, reduce surface free energy, and block heterogeneous nucleation processes	Clinical	Can significantly reduce the formation of inorganic and organic deposits	Limited effectiveness against existing biofilms or encrustations
Antimicrobial strategies	Bacterial adhesion and biofilm formation	Antimicrobial peptides, quaternary ammonium salts, and silver ions	Disrupt the cell membrane structure, induce oxidative stress, and inhibit quorum sensing (QS) signaling	Clinical	Can directly reduce the risk of urinary tract infections	Potential risks of cytotoxicity and long-term resistance
Anti-inflammatory strategies	Inflammatory response and immune activation	Dexamethasone and ketorolac	Inhibition of NF-κB, MAPK, and related inflammatory signaling pathways	Animal/*in vitro*	Improves tissue compatibility and enhances long-term tolerance	No direct inhibitory effect on bacterial colonization or infection
Antifibrotic strategies	UMT and ECM deposition	TGF-β inhibitors and RNA interference systems	Blocking the TGF-β/Smad signaling pathway to inhibit myofibroblast activation and excessive ECM deposition	Animal/*in vitro*	Might decrease the likelihood of ureteral stricture and fibrotic changes	Most are still in the experimental research stage, with limited clinical translation
Multifunctional synergistic strategies	Holistic regulation of the microenvironment (infection–encrustation–inflammation coupling)	Zwitterionic polymer-based antimicrobial composite coatings, drug-eluting systems, and multilayer structural coatings	Synergistically regulate protein adsorption, bacterial colonization, and inflammatory responses to achieve multitarget intervention	Animal/*in vitro*	Better suited to the demands of complex clinical microenvironments	Complex manufacturing process with high stability and regulatory requirements
Smart response strategies	Dynamic changes in the pathological microenvironment (pH, ROS, enzymes, temperature, etc.)	pH/ROS-responsive polymers, hydrogel drug delivery systems, stimulus-responsive nanocoatings, and dynamically bonded materials	Trigger structural changes or drug release in infectious or inflammatory microenvironments (acidic, high ROS, enzyme activation, etc.) to achieve “on-demand release” and interface-adaptive regulation	Animal/*in vitro*	Enables dynamic, spatiotemporally controlled therapy, improving precision and efficiency while reducing systemic side effects	System design is complex, with high demands on stability, response thresholds, and long-term reliability; clinical validation is insufficient

### 4.2 Antimicrobial strategies

Microbial infections, including *Escherichia coli*, *Proteus mirabilis*, and *Enterococcus faecalis*, are prevalent in the urinary tract. Biofilm formation occurs when bacteria cling to the stent surface. The biofilm’s distinctive architecture fosters bacterial proliferation, making the bacteria resistant to standard antibiotics (Hou et al., 2022). Consequently, surmounting infection and the development of bacterial biofilms presents a significant challenge for researchers. Presently, often utilized antimicrobial coatings for ureteral stents comprise cationic polymers, antimicrobial peptides (AMPs), metal ions, among others.

#### 4.2.1 Cationic polymer coatings

Cationic polymers exert contact-based antibacterial activity by electrostatically adhering to the negatively charged bacterial membranes through their high-density positive surface charges, resulting in membrane breakdown. This technique does not emit bactericidal chemicals, therefore circumventing the possible detrimental effects associated with conventional antibacterial approaches, such as bacterial resistance and cytotoxicity (Gupta and Malgar Puttaiahgowda, 2022).

Quaternary ammonium salt (QAS) cationic polymers are the optimal selection for formulating contact-killing coatings. Research indicates that QAS small molecules with extended chains (C10–C14) demonstrate significant antibacterial efficacy (Nunes et al., 2023). Pan et al. (2024) synthesized SI-QAS antimicrobial coatings by chemically grafting polyurethane intermediates with varying concentrations of QAS onto the surface of a pretreatment vinyl-rich silica layer (SI-vinyl). Experiments demonstrated that the SI-QAS-2 scaffold possesses a surface QAS concentration of 2.1 × 10^16^ N^+^/cm^2^ and achieved bactericidal rates of 72.8%, 99.9%, and 98.9% against *Escherichia coli*, *Staphylococcus aureus*, and *Pseudomonas aeruginosa*, respectively, within 30 min and sustained over 80% antimicrobial activity for 4 days in a high-concentration bacterial environment.

Cationic antimicrobial coatings may be integrated with hydration layers and degradable functional groups to create multifunctional stents. Li et al. (2022) synthesized the macromolecule P (LA-co-AC), which possesses primary amine groups in its side chains. This compound can compromise bacterial cell membranes by imitating antimicrobial peptides. Combining P (LA-co-AC) with the PLACL matrix in a defined ratio produces an innovative biodegradable ureteral stent exhibiting antibacterial characteristics. Experimental findings indicate remarkable bacteriostatic and bactericidal efficacy against *Escherichia coli*. Also, an increase in the concentration of P (LA-co-AC) correlates with a substantial reduction in the number of free bacterial colonies.

Cationic antimicrobial scaffolds have benefits, including rapid antimicrobial efficacy, extensive antimicrobial ranges, and minimal chance of resistance emergence, rendering them appropriate for short-to-medium-term indwelling catheters, recurrent urinary tract infections, and high-risk demographics. *In vivo*, bacterial fragments can bind to the positively charged antimicrobial surface by electrostatic attraction, leading to a biofilm that obscures the bactericidal groups, provides a surface for attachment of neighboring free-floating bacteria, and ultimately reduces the bactericidal potency of surface contact. The “biofilm masking effect” has become an important challenge that must be addressed immediately in the research and development of cationic antimicrobial stents.

#### 4.2.2 Antimicrobial peptide coatings

AMPs exhibit a unique architecture of amphiphilic residues and cationic characteristics. Unlike antibiotics, they act on the lipopolysaccharide layer of the cell membrane and thereby affect the membrane integrity and enable contact-mediated bactericidal activity. Therefore, AMPs have an immense potential for infection reduction, inspired by mussels. (Yao et al., 2022a) developed two antibacterial approaches for the functionalization of polyurethane ureteral stents. A composite coating (PDPA@Cu-75–AMP) was synthesized based on Cu^2+^-assisted polydopamine coordination and the covalent grafting of antimicrobial peptides (Yao et al., 2022a). *In vitro* studies showed that this coating could inhibit bacterial growth for 14 days and significantly reduce stent biofouling by 77%. Later, Cu^2+^-coordinated clickable mussel peptides were used with AMPs to develop a stable coating, which improved click-mediated assembly and enhanced its structure in a multifunctional manner, systematically optimizing antimicrobial efficacy, grafting density, and stability (Yao et al., 2022b). Both techniques use a mussel-inspired mechanism to promote the interfacial adhesion, and Cu^2+^ coordination can effectively enhance the coating durability. The *in vitro* tests showed that both modified scaffolds attained bactericidal rates close to 100% against *Escherichia coli*, *Staphylococcus aureus*, and *Proteus mirabilis*. The two research projects reported here extend the use of antimicrobial peptides to medical implants and lay a technical foundation for the anti-infective modification of more implantable devices. AMPs have the advantages of higher bactericidal efficacy and lower probability of resistance development and biocompatibility and are very promising for the future development of ureteral stents.

#### 4.2.3 Metal-ion coatings

Metal-ion coatings are a traditional approach, showing broad-spectrum antimicrobial effects through mechanisms including disturbance of bacterial cell membrane integrity, bringing about oxidative stress, and interference with DNA replication (Fosca et al., 2023). Nonetheless, with the continuous development of coating technology, researchers have found that a single metal-ion coating cannot completely prevent stone formation and bacterial infections. Consequently, new coatings that combine metal ions with other functional components have become a hot topic.

Rodriguez-Alvarez et al. (2026) used glycol-chitosan-stabilized silver nanoparticles on a hydroxylated fullerene scaffold and applied them to a polyurethane surface using dip-coating. This coating accomplished total eradication of six clinically harmful urinary tract bacteria and sustained significant antibacterial activity for four consecutive days, leading to a coated surface entirely devoid of biofilm and bacterial adherence. Zhai et al. (2025) cross-linked zinc ions with antibacterial polyacrylic acid (PAA(Zn(2+)) and integrated it with polyvinylpyrrolidone (PVP), known for its hydrophilic lubricating qualities, applying the resultant material to a PU scaffold (PPAZ/PU). This coating imparts the scaffold with significant antibacterial qualities, superlubricity, and efficient antifouling capabilities through a synergistic approach that includes the development of a hydration layer and the loss of bacterial cell membrane integrity by Zn^2+^. The coating has antibacterial efficiency surpassing 99% against *Escherichia coli*, *Staphylococcus aureus*, and *Proteus mirabilis* while decreasing fouling rates by roughly 75%.

Metal-ion-based stent coatings have benefits, including extensive antibacterial efficacy and prolonged, steady release, and can be integrated with diverse functional elements to produce multifunctional coatings. Nonetheless, these coatings possess drawbacks, such as possible cytotoxicity at elevated doses, the possibility of localized inflammation, and inadequate long-term release stability.

### 4.3 Anti-inflammatory and antifibrotic strategies

Chronic inflammatory reactions and fibrosis induced by stent implantation are the principal pathogenic mechanisms responsible for ureteral strictures and tissue destruction. Therefore, regulating the local immune microenvironment through the incorporation of anti-inflammatory drugs or immunomodulatory agents may provide a promising strategy for mitigating these pathological processes. Conventional anti-inflammatory and antifibrotic strategies are primarily based on systemic administration or passive release of anti-inflammatory drugs from coatings. However, these methods suffer from low drug bioavailability, insufficient local concentration, and systemic side effects. Recently, important advances have been made in the study of modulation of inflammatory signaling pathways and fibrotic processes by local drug delivery systems.

Present research is concentrating on antifibrotic coatings based on mTOR inhibitors. Meng et al. (2025) developed a TGF-β1-siRNA stent with sustained release of poly(lactic-co-glycolic acid) (PLGA)-siRNA particles that can deliver siRNA to selectively target TGF-β1 mRNA, leading to a significant decrease in TGF-β1 protein production and inhibition of the TGF-β1/Smad signaling pathway. In the rabbit model, the siRNA scaffold group had a significant decrease in strictures, and hydronephrosis was significantly relieved, which effectively prevented the ureteral strictures. Rapamycin (RAPA) is a macrolide immunosuppressant shown to possess antifibrotic properties. This drug inhibits the activity of mTORC1, which subsequently inhibits the TGF-β/Smad-mediated epithelial–mesenchymal transition and excessive collagen deposition, reducing the fibrotic strictures after ureteral injury, making it a commonly used antifibrotic treatment (Zhang et al., 2022). Duan et al. (2025) combined paclitaxel (PTX) with rapamycin (RAPA), which inhibits excessive collagen accumulation for dual antistricture effects. In this study, a three-layer ureteral stent with antistricture properties was fabricated, and the fibroblast inhibition rate was 41.78%, which greatly inhibited cell proliferation (Duan et al., 2025). Later, Duan et al. (2024) developed a degradable poly(p-dioxanone) (PDO) scaffold-silk fibroin (SF) drug-eluting stent, loaded simultaneously with RAPA and PTX. *In vitro* tests revealed that the high-dose dual-drug group exhibited significantly higher inhibition rates of fibroblasts and smooth muscle cells than the single-drug treatment. The stents in rats maintained continuous luminal patency for 7–28 days postimplantation and demonstrated biodegradable properties, offering a new method for clinical antistenosis therapy. Pirfenidone is an oral antifibrotic agent that can inhibit the TGF-β/Smad signaling pathway by downregulating the expression of TGF-β1 and TGF-β3, thereby blocking fibroblast proliferation and reducing the formation of the extracellular matrix. It has broad antifibrotic and anti-inflammatory effects (Li N et al., 2022). Cai et al. (2023) developed a double-layer drug-eluting ureteral stent based on pirfenidone, polydopamine, and hyaluronic acid. The stent’s unique design with an inner polydopamine layer and an outer hyaluronic acid layer allows for rapid drug release during the first 5 days, followed by slow release from days 6 to 14, covering the entire critical period of ureteral stricture repair. In the minipig ureteral stricture model, the patency of the ureter was preserved in the drug-eluting stent group at 6 weeks postoperatively without significant stricture or occlusion. Tian et al. (2025) designed a ureteral stent coated with a composite of pirfenidone-loaded PLGA nanoparticles and polyacrylamide hydrogel. The hydrogel was tightly bound to the stent by UV-induced *in situ* polymerization, which allowed controlled drug release up to 12 weeks. *In vitro* studies demonstrated that it significantly inhibited fibroblast proliferation and collagen synthesis.

The major mechanism of action of the anti-inflammatory/antifibrotic scaffold is the accurate targeting of the inflammation–fibrosis cascade. These stents locally deliver pharmaceuticals at the site of injury to prevent critical pro-inflammatory and profibrotic signaling pathways such as mTOR, NF-κB, and TGF-β/Smad; downregulate the release of pro-inflammatory mediators such as IL-1β, IL-6, and TNF-α; block fibroblast transformation into myofibroblasts; and limit excessive production and deposition of the cell-external matrix. Stents use biodegradable polymers as a carrier to allow a sustained and uniform release of the drug and to reduce the side effects of the systemic drug. They show good biocompatibility and fewer foreign body responses. However, they present disadvantages of a short drug release period, difficulty in precisely matching the degradation rate with the tissue repair cycle, complicated production processes, and relatively high costs.

### 4.4 Stimuli-responsive functionalized stents

Smart-responsive coatings can sense variations in the external or local microenvironment (e.g., pH, temperature, or enzymatic stimulation) and respond adaptively to pathological conditions (Pereira et al., 2026). Their structure or characteristics can be modified to allow controlled drug release, surface property modification, or functional activation. In other fields of medical devices, such as vascular stents, orthopedic implants, and urinary catheters, intelligent response coatings were developed earlier with effective anti-infective, antifouling, and tissue repair properties (Hu Q et al., 2024; Zhu et al., 2025). However, smart-responsive coatings for ureteral stents are still at the early exploration stage.

Smart-responsive coatings can respond to cues such as urease, pH changes, or increased levels of reactive oxygen species (ROS) in the milieu of infection or inflammation by triggering the localized release of pharmaceuticals, thus enabling proactive prevention and treatment of infection, scaling, and fibrosis. Li et al. (2024) introduced a urease-responsive smart lubricating coating on the basis of a pH-responsive polymer that can trigger the pulsed release of antimicrobial agents in the alkaline microenvironment (pH > 8.0) caused by urease-producing bacterial infections, which can ensure sufficient drug exposure at the infection stage but avoid redundant release at normal pH conditions. Then, Jia and Zhang (2025) proposed a drug delivery approach in which the antibacterial agent sulfamethoxazole was encapsulated in chemical bonds using urease-sensitive polymers. Drug release is triggered only in the alkaline environment (pH > 8) provided by urease-positive bacterial infection, thus enabling a bactericidal activity on demand. In addition to the above conventional chemical-type smart coatings, physical signals such as pressure can also be used to enable real-time monitoring and early warning, and such coatings are classified as physical-type smart responses. A sophisticated ureteral stent with wireless pressure sensing and remote monitoring capabilities was reported by YousefiDarestani (2024), which allows for the early diagnosis of pathological conditions such as hydronephrosis by continuous monitoring of intrapelvic pressure.

Besides the aforementioned smart-responsive coatings, reactive oxygen species (ROS) are also important factors in the regulation of stent-related inflammation, NOD-, LRR-, and pyrin domain-containing protein 3 (NLRP3) inflammasome activation, and fibrosis (Wang et al., 2020). Thus, ROS-responsive drug delivery systems that utilize the elevated ROS level in the inflammatory microenvironment to activate ROS-sensitive chemical bonds for controlled drug release have great application potential (Gao and Xiong, 2021). Alternative thermoresponsive methodologies mainly exploit the properties of thermosensitive polymers like reversible phase transitions, swelling and shrinking, or structural changes at certain temperatures to achieve controlled drug release or surface functional modulation (Kobayashi et al., 2025). From a theoretical point of view, stent-related infection and inflammation can cause a slight increase in local temperature, which in turn leads to drug release from the thermosensitive coating. However, because local temperature changes in the urinary tract exhibit limited range and poor stability, temperature lacks the requisite specificity and sensitivity to serve as an independent triggering signal. Therefore, future applications may favor a multistimulus synergistic response system by combining temperature responsiveness with pH, ROS, or urease responsiveness, which can enhance the ability of the ureteral stent to detect the pathological microenvironment and improve therapeutic precision.

## 5 Current challenges and future perspectives in clinical translation

### 5.1 Current status

Recently, clinical research on ureteral stents has progressively transitioned from conventional material comparisons to functional enhancements, structural optimization, and biodegradable stent innovation. According to current clinical evidence, the stents that have progressed to human trials predominantly consist of drug-eluting stents, anti-encrustation-coated stents, material-optimized stents, structurally optimized stents, and biodegradable stents. Material-optimized and structurally optimized stents have garnered substantial evidence from randomized controlled trials, but functionalized stents exhibiting antibacterial, anti-inflammatory, and antifibrotic capabilities are predominant in the first phases of clinical validation (Table 3).

**TABLE 3:** Clinical trials of ureteral stents.

Classification	Strategy/Stent	Year	Study design	Sample size	Main research content	Main results
Biodegradable scaffold	TUDS Phase II	2003	Prospective study	88 patients	Evaluation of TUDS efficacy	71 of 80 patients (89%) were satisfied with TUDS, as it merges effective ureteral drainage with patient satisfaction, thus eliminating the need for simple ureteroscopy and stent removal (Lingeman et al., 2003a)
Biodegradable scaffold	TUDS Phase I	2003	Prospective study	18 patients	Evaluation of TUDS durability, patency, and safety	TUDS facilitated ureteral drainage for at least 48 h after placement and demonstrated good safety characteristics (Lingeman et al., 2003b)
Antimicrobial-coated stents	Triumph® triclosan-eluting stent	2012	Randomized controlled trial (RCT)	20 patients	To determine the effectiveness of ureteral stents loaded with triclosan in decreasing bacterial adhesion, biofilm formation, and encrustation formation linked to stents	The Triumph® triclosan-eluting stent showed no significant difference in terms of biofilm and scabbing, but there was a significant reduction in lower abdominal pain among subjects (Mendez-Probst et al., 2012)
Antimicrobial-coated stents	Silver sulfadiazine (SSD)-coated stent	2018	Randomized controlled trial (RCT)	126 patients	Comparison of the efficacy of antimicrobial-coated ureteral stents versus uncoated stents in preventing stent-associated bacteriuria (El-Nahas et al.)	In the group with stents coated with silver sulfadiazine (SSD), bacteriuria occurred less often than in the control group, but this difference did not reach statistical significance (p = 0.372) (El-Nahas et al., 2018)
Anti-encrustation-coated stents	Hydrogenated-coated silicone stent	2021	Prospective randomized controlled trial	141 patients	Evaluation of hydrogenated-coated silicone stents versus Percuflex™ Plus stents in terms of biofilm formation and encrustation	Compared with Percuflex™ Plus stents, silicone water-in-oil stents showed less encrustation after being in the hospital for 3 weeks (Barghouthy et al., 2021)
Anti-encrustation-coated stents	Tria™/PercuShield™-coated stents	2025	Prospective study	359 patients	A combined comparison of Tria stents and non-Tria stents evaluated technical success rates, complications, and patient symptoms	Tria™ stents significantly improved patients’ pain interference scores and increased comfort during placement; however, no significant advantages were observed in terms of infection rates or encrustation rates (Ren et al., 2025)
Structurally optimized stents	Single-tail-suture stent (SPSS)	2020	Prospective randomized controlled trial	130 patients	Evaluation of the tolerability of single-tail suture stents (SPSS) following uncomplicated retrograde semirigid ureteroscopic lithotripsy (URSL)	In addition to sexual function indicators and general health index scores, the SPSS was effective across all domains of the Ureteral Stent Symptom Questionnaire (USSQ); patients treated with SPSS had significantly better quality-of-life scores (Bostanci et al., 2020)
Structurally optimized stents	Double-tail suture stent (PSS)	2021	Prospective randomized controlled trial	78 patients	To determine whether commercially available double-tailed suture stents (PSS) can alleviate stent-related symptoms compared with conventional DJ stents	In comparison with standard DJ stents, PSS significantly lessened symptoms related to stents, particularly urinary symptoms and pain, after URS, while maintaining good safety (Bosio et al., 2021)
Structurally optimized stents	Antireflux stent	2023	Randomized controlled trial (RCT)	120 patients	Comparison of the efficacy of antireflux ureteral stents in improving symptoms and quality of life in patients treated with ureteral stents	Antireflux ureteral stents offer equivalent safety and efficacy to conventional ureteral stents, but they are significantly superior to standard ureteral stents in terms of quality of life, including lateral abdominal pain and suprapubic pain (Zhang et al., 2023)
Structurally optimized stents	Retrograde placement DJUS	2025	Prospective randomized controlled trial	120 patients	To assess the clinical benefits and patient discomfort reported with standard DJUS placement versus the retrograde technique	Placing the DJUS in reverse, especially with both stent ends positioned laterally, might lessen discomfort related to the stent (Bayar et al., 2025)
Structurally optimized stents	Silicone-coated metal ureteral stents	2026	Randomized controlled trial (RCT)	65 patients	To assess the effectiveness and safety of silicone-coated metal stents compared with double-J stents in patients with malignant ureteral obstruction	Silicone-coated metal stents outperformed double-J stents in terms of maintaining urine flow and overall quality-of-life scores, with no significant differences in safety or patient satisfaction (Han et al., 2026)
Magnetic stents	Magnitip DJ	2019	Prospective randomized controlled trial	50 patients	To analyze the rates of complications associated with the Magnitip double-J stent versus standard stents and to investigate the potential for magnetic tip retrieval of the DJ stent without the need for cystoscopy	There was a higher rate of complications linked to magnetic DJ stents, but their removal cost was less than that of standard DJ stents (Farouk et al., 2019)
Magnetic stents	Black-Star Magnetic Stent	2021	Prospective randomized controlled trial	41 patients	Comparison of results concerning clinical and economic factors between conventional and magnetic stents in patients who received kidney transplants from deceased donors	The USSQ scores indicated that tolerance during removal of the Black-Star magnetic stent was comparable to that observed with flexible cystoscopy in kidney transplant patients. The Black-Star stent was removed significantly faster than conventional stents. No significant differences were observed in discomfort, infection rates, or complication rates (Kapoor et al., 2021)
Material-optimized stents	Polaris Ultra HydroPlus™ vs. Tria™ PercuShield™	2021	Prospective randomized controlled trial	84 patients	Comparison of short-term slough formation outcomes between the Polaris Ultra HydroPlus™ and Tria™ PercuShield™ stents	In the short term, the Tria and Polaris Ultra stents were equally effective in preventing crusting (Yoshida et al., 2021)
Material-optimized stents	Universa® Soft Stent vs. Percuflex® Rigid Stent	2026	Randomized controlled trial (RCT)	120 patients	Both soft- and hard-polymer stent groups were evaluated using the Ureteral Stent Symptom Questionnaire (USSQ)	The use of soft ureteral stents compared with rigid stents reduces stent-related symptoms, provides a superior quality of life, and maintains comparable safety (Hemo et al., 2026)

Material optimization is presently one of the most advanced clinical translation pathways. A multicenter, randomized, single-blind research study by Barghouthy et al. (2021) involved 141 patients with ureteroscopic lithotripsy to compare a silicone hydrogel-coated stent (Imajin® Hydro) with the Percuflex™ Plus stent. The findings indicated that the silicone stent had reduced biofilm development and mineral deposition scores following a 3-week insertion period, implying that the surface characteristics of the material can substantially affect encrustation and biofilm formation. Zhang et al. (2023) performed a study using random allocation and control conditions on 120 patients with ureteral stones to compare antireflux stents and conventional double-J stents. The study showed that antireflux stents significantly relieved lower back pain and bladder irritation symptoms and improved the quality of life of patients. There were no substantial differences in the infection rate and hydronephrosis between antireflux stents and conventional stents.

Functionalized stents generally obtain antibacterial, anti-encrustation, and anti-inflammatory properties through drug elution or surface modification. Clinical trials have been initiated for triclosan-eluting, ketoprofen-eluting, and ketorolac-loaded ureteral stents (Joshi et al., 2010; Mendez-Probst et al., 2012). However, most studies have shown that these stents did not demonstrate significant therapeutic advantages in terms of infection rates, biofilm formation, and encrustation control, with only marginal advantages in terms of pain relief or symptomatic improvement. The lack of statistical significance for specific endpoints in the clinical trials mentioned above may be due to the use of small-sample pilot studies; alternatively, in larger multicenter trials, the effective sample size may still be inadequate after subgroup analyses of low-incidence clinical endpoints. Moreover, the documentation of the statistical power analyses in these studies was often poor, especially for adverse outcomes such as infection, stent calcification, and pain management procedures, with prespecified clinically significant differences, the power of the test needed, and the sample size needed often not being adequately detailed.

The anti-encrustation property is another class of functionalized stents with significant clinical relevance. Heparin coating can prevent calcification by blocking crystal adhesion and mineralized deposit. In a clinical investigation, heparin-coated stents were demonstrated to significantly reduce calcification thickness and biofilm formation after short-term implantation (Cauda et al., 2008). Long-term stent implantation in heparin-coated stent surfaces showed negligible calcification and remarkable and sustained anticalcification efficacy. Tria™, a recently designed multilayer stent with an anti-encrustation coating, incorporates a design on both its inner and outer surfaces that enhances patient comfort by maximizing surface lubricity. In a prospective analysis of 359 patients, it failed to attain statistical significance in critical outcomes, including the reduction of infection incidence and the minimization of encrustation formation (Ren et al., 2025). The results indicate that dependence exclusively on anti-encrustation techniques may be inadequate to concurrently mitigate stent-related problems caused by many variables, including infection and inflammation.

Biodegradable ureteral stents represent a very promising domain for future advancement. The temporary ureteral drainage stent (TUDS) is presently the sole biodegradable ureteral stent that has finalized a prospective clinical trial. Phase I clinical trials showed its good short-term drug effectiveness and safety (Lingeman et al., 2003b). Later Phase II clinical trials confirmed its ability to maintain efficient drainage and to achieve high patient satisfaction (Lingeman et al., 2003a). Clinical tests of the TUDS series proved that it could guarantee sufficient drainage and avoid re-operation, which makes patients’ treatment more comfortable. However, challenges such as precisely controlling degradation kinetics, the potential accumulation of degradation by-products, and the long-term biosafety of degradation derivatives remain to be addressed (Hu K et al., 2024). For clinical use, biodegradable ureteral stents should have consistent degradation characteristics and safely remove degradation products. Clinical studies are presently being conducted on the Uriprene® and Hydrustent® biodegradable ureteral stents to assess their safety and feasibility for efficient drainage, stent degrading integrity, and patient tolerance.

### 5.2 Challenges and future perspectives

Recently, various functionalization methods and intelligent response technologies have been verified by *in vitro* studies and animal models, with promising results in improving the antimicrobial, antiscaling, anti-inflammatory, and tissue-protective properties of ureteral stents. However, the subsequent application of these techniques in clinical practice still faces considerable challenges. First, *in vitro* models and animal studies have difficulty accurately reproducing the complex and dynamic microenvironment of the human urinary system, including changes in urine composition, hydrodynamic shifts, bacterial community structures, and host immune responses. The regional pathological microenvironment that occurs after ureteral stent placement is highly dynamic and varies greatly between patients, which may explain why some functionalized stents fail to perform in clinical settings despite their success in experimental models.

Second, the functionalized coatings and surface modification methods still suffer from the problems of stability and durability during long-term implantation. The stent surface is continuously exposed to the urinary environment and is affected by multiple factors, including mechanical abrasion, chemical degradation, microbial colonization, and ongoing inflammatory reactions. This exposure can result in degradation, displacement, or diminished performance of the active coating. The degradation of biodegradable ureteral stents may generate unexpected debris, residuals, and by-products, which might affect the local microenvironment of the urinary tract and the safety of the stent. The size, form, and rate of degradation fragments may influence their elimination by urine. Larger fragments can cause obstruction of the urinary tract, damage of the mucosa, or local mechanical irritation. Partial degradation of some stent materials may result in residual polymers or non-degraded components persisting in the ureteral wall for long periods, which may lead to a chronic foreign body response or failure of tissue regeneration. Furthermore, small-molecule products of degradation may alter the local urinary milieu, resulting in an influx of inflammatory cells and release of inflammatory mediators, which may lead to fibrosis or ureteral constriction. Thus, controlled degradation, safe elimination of degradation products, and long-term biocompatibility are the key challenges to be tackled in the future development of biodegradable stents.

In addition, sophisticated manufacturing techniques and material frameworks have limited the large-scale manufacturing, quality control, and clinical application of functionalized ureteral stents. The assessment metrics, experimental parameters, and clinical outcomes are very different between studies. In addition, the absence of large, multicenter, and long-term follow-up studies prevents a complete assessment of the real clinical benefits of many new stent technologies at present. Therefore, functional ureteral stents need to be translated from experimental studies to clinical applications, requiring a uniform performance evaluation paradigm and an ongoing clinical validation system.

Upcoming research efforts need to be directed toward the systematic analysis of the processes of the ureteral stent microenvironment and encourage the deep integration of multifunctional integration strategies with a smart-responsive design to achieve the exact regulation of the pathological microenvironment. In parallel, the optimization of the coating formulations and stent fabrication procedures and the setting up of a consistent long-term clinical evaluation system are needed. Such multilevel and coordinated enhancements are projected to result in the substantial transformation of ureteral stents from typical protective devices to smart devices capable of active regulation and precise intervention.

## 6 Conclusion

The review provides an overview of the mechanisms involved in the pathological microenvironment associated with ureteral stents, the emergence of major complications related to ureteral stents, and the latest advancements in surface modification approaches for ureteral stents. To enhance the results after ureteral stenting and minimize stent-related complications, a number of advances in stent development have been made by researchers via strategies such as material upgrades and surface coating development. However, with the deepening of understanding of the stent microenvironment, researchers found that the occurrence of stent-related complications is not due to a single factor. Rather, it is the continuous interaction of the complex pathological microenvironment induced after stent implantation and the host response. These key processes include biofilm formation, crystal deposition, activation of inflammatory responses, and fibrotic remodeling. A comprehensive analysis of these pathogenic mechanisms and their dynamic regulatory networks helps overcome the limitations of traditional ureteral stents, which solely depend on passive drainage and mechanical support. It will also guide the design and development of functionalized ureteral stents that can actively regulate the microenvironment, thereby providing new strategies to reduce clinical complications and improve treatment benefits for patients.

Recently, based on the concept of pathological microenvironment regulation, the functionalization of ureteral stent surfaces has developed rapidly, with functional directions such as antiscaling, antibacterial, anti-inflammatory, and antifibrotic properties. These strategies have gradually developed from single-function optimization to synergistic integration of multifunctional capabilities and smart, responsive regulation. However, most of the functionalization technologies are still in the stage of preclinical research, and limited clinical studies have not yet fully revealed their long-term benefits. However, the clinical translation of functionalized stents faces several challenges, including long-term stability, biosafety, large-scale manufacturing, and insufficient clinical validation. The development of ureteral stents should further promote the integration of multifunctional materials and smart-responsive systems in the future so that stents can achieve accurate dynamic regulation in response to the changes of the urinary tract microenvironment. Meanwhile, well-designed clinical studies with adequate sample size, standardized evaluation systems, and long-term follow-up are required to clarify the real clinical benefits of these emerging technologies. In the long run, the transition from traditional passive drainage devices to actively regulated smart ureteral stents is anticipated to be a significant development trend in reducing stent-related complications and enhancing patients’ treatment experience and long-term prognosis.
