# Supplementary material for: From biofilms to fibrosis: recent advances in research on the pathological microenvironment linked to ureteral stents and their surface functionalization
Source: Front Bioeng Biotechnol. 2026 Sep 28;14:1925047. doi: 10.3389/fbioe.2026.1925047 (PMC13655537; doi:10.3389/fbioe.2026.1925047)
Supplement: Supplementary file 1 [file Table1.docx]

Supplementary table 1 Figure 3 legend

| **Abbreviation** | **Full name** |
| --- | --- |
| TLR2/4 | Toll-like receptor 2/4 |
| P2X7R | P2X purinoceptor 7 |
| PAMPs | Pathogen-associated molecular patterns |
| IL-6 | Interleukin-6 |
| TGF-β | Transforming growth factor beta |
| MyD88 | Myeloid differentiation primary response 88 |
| IKKα/β/γ | Inhibitor of nuclear factor kappa-B kinase α/β/γ |
| IκBα | Inhibitor of nuclear factor kappa B alpha |
| NF-κB | Nuclear factor kappa B |
| MAPK | Mitogen-activated protein kinase |
| ERK1/2 | Extracellular signal-regulated kinase 1/2 |
| p38 MAPK | p38 mitogen-activated protein kinase |
| JNK | c-Jun N-terminal kinase |
| AP-1 | Activator protein-1 |
| NLRP3 | NOD-, LRR- and pyrin domain-containing protein 3 |
| ASC | Apoptosis-associated speck-like protein containing a caspase recruitment domain |
| JAK/STAT | Janus kinase/signal transducer and activator of transcription pathway |
| Smad | Mothers against decapentaplegic homolog proteins |
| PI3K | Phosphoinositide 3-kinase |
| Akt | Protein kinase B |
| ROCK | Rho-associated coiled-coil-containing protein kinase |
| c-Fos | Fos proto-oncogene |
| c-Jun | Jun proto-oncogene |
| mTOR | Mechanistic target of rapamycin |
| MLC | Myosin light chain |
